# Test for clinical reasoning evaluation in Speech-Language Pathology: content validity

**DOI:** 10.1590/2317-1782/20242023276en

**Published:** 2024-05-31

**Authors:** Ana Cristina Côrtes Gama, Aline Mansueto Mourão, Adriane Mesquita Medeiros, Patrícia Cotta Mancini, Thais Helena Machado, Lara Gama Santos, Nayara Ribeiro Gomes

**Affiliations:** 1 Departamento de Fonoaudiologia, Faculdade de Medicina, Universidade Federal de Minas Gerais – UFMG - Belo Horizonte (MG), Brasil.; 2 Centro Federal de Educação Tecnológica de Minas Gerais – CEFET - Belo Horizonte (MG), Brasil.; 3 Programa de Pós-graduação (doutorado) em Ciências Fonoaudiológicas, Departamento de Fonoaudiologia, Faculdade de Medicina, Universidade Federal de Minas Gerais – UFMG - Belo Horizonte (MG), Brasil.

**Keywords:** Speech-Language Pathology, Clinical Reasoning, Clinical Diagnosis, Clinical Decision-Making, Students, Learning

## Abstract

**Purpose:**

To validate the content of the Speech-Language Pathology Concordance Test called FonoTCS.

**Methods:**

This is a content validation study of the instrument. Five speech-language pathologists, all with doctoral degrees and teaching experience, averaging 24.8 years of professional practice, participated in the development of FonoTCS and reached a consensus during the process. Thirty questions and 120 items were created, covering seven areas of speech-language pathology expertise across three domains. For content validation, FonoTCS was electronically sent to 15 evaluators to respond to a questionnaire with five questions, rated on a five-point scale, regarding the criteria of clarity, ethics, and relevance of the questions. The Corrected Content Validity Coefficient was calculated for all statements to analyze the responses. Questions with agreement percentages equal to or less than 80% were revised.

**Results:**

Thirteen evaluators, all female, with an average age of 39.07 years, including eight with master's degrees and five with doctoral degrees, and an average clinical practice experience of 15.38 years, participated in the analysis. The average Corrected Content Validity Coefficient values for the clarity criterion were 0.93 and 0.95, for the relevance criterion 0.98 and 0.92, and for the ethics criterion 0.99. Two questions received scores of 0.78 and 0.80, both related to the audiology area in the assessment/diagnosis domain, specifically question 2 regarding the relevance criterion. These questions were reviewed and restructured by the judges.

**Conclusion:**

FonoTCS is a valid instrument from a content perspective.

## INTRODUCTION

Clinical reasoning is a cognitive process that allows the health professional to define a correct diagnosis and an appropriate therapeutic approach in each clinical case^(1)^. The cognitive process involves mental elaborations linked to the care of users of health systems. It is a central topic in the health education area and the exercise of professional practice^(2)^. Currently, one of the accepted theories for clinical reasoning considers the development of mental scripts^(3-6)^.

In this theory, through repetitive exposure to clinical cases, health professionals create mental schemas of certain clinical conditions, called “disease scripts”, which would be stored in memory^(7)^. A script would represent a network of specific knowledge, where multiple elements of information are organized according to their relationships^(6)^.

Experienced professionals have elaborate networks of knowledge linked to the diagnostic or therapeutic decision process, that is, refined mental scripts, which provide assertive clinical reasoning^(7)^. The scripts are made by correlating information about disorders or diseases, their clinical characteristics, and treatment possibilities^(6,8)^.

According to the National Curricular Guidelines for the Undergraduate Course in Speech-Language Therapy (CNE/CES 5)^(9)^, one of the skills necessary to practice the profession is the ability to make decisions for speech-language therapy practices, which involve the correct diagnosis and definition of the model most appropriate intervention method for each clinical problem^(9)^.

Learning strategies for developing clinical reasoning^(10)^ and ways to evaluate and monitor the progress of health students' performance^(11)^ are described.

Difficulties in clinical reasoning among speech-language therapy students when making diagnoses^(5)^ and important differences in clinical reasoning between students and experienced professionals^(12)^ are highlighted. The challenge of how to evaluate students' clinical reasoning performance during their academic training is constant in health curricula, including speech-language therapy^(13)^.

The Script Concordance Test (SCT) is based on the principle that multiple judgments made in the clinical reasoning process can be investigated, and their agreement with those of a panel of reference experts can be measured^(14,15)^.

The SCT was developed to assess reasoning in uncertain situations^(16)^ that frequently occur in daily practice, especially in healthcare professionals^(14)^. Guidelines for the preparation of the SCT^(14-16)^, which explain rules for the preparation and administration of protocols, were proposed so that these instruments are reliable and valid. The principle of the test is to be based on clinical cases that must be described in short scenarios and always incorporate uncertainty^(15)^.

The guidelines for the construction of the SCT have some recommendations^(14-16)^ such as the number of cases; the number of members to prepare the instrument; the content validity analysis; the definition of the test score using the aggregated score method; and the presentation in electronic format to present imaging exams and return the results to the examined users.

A series of studies on SCT have been developed in medicine^(17)^, nursing^(18)^, dentistry^(19)^, veterinary^(20)^, and physiotherapy^(21)^. The results pointed to the validity of the SCT and its ability to differentiate reasoning depending on the degree of professional experience^(13,17-19)^. In Brazil, the SCT was developed to evaluate the clinical reasoning of medical students in clinical situations in geriatrics^(22)^, and nursing^(23)^. We did not find studies in the literature that propose SCT in speech-language therapy.

Assessment instruments play a crucial role in obtaining information. However, these tools must have psychometric attributes such as validity and reliability to ensure confidence in the evaluated indicators^(24)^. The Joint Committee on Standards for Educational and Psychological Testing^(25)^ presents five sources of valid evidence: (1) content; (2) internal structure; (3) relationship with external measures; (4) response pattern to items; and (5) consequential^(25)^.

Considering that instruments for assessing clinical reasoning are incipient, that the SCT has been validated for students from different areas^(17-21)^, and that for speech-language therapy there is still no research on the SCT, this project aims to validate the content of the Script Agreement Test in Speech-Language Therapy, called *FonoTCS (for its Portuguese acronym)*.

Based on the results of this research, the *FonoTCS* will move on to validating the internal structure and developing its virtual format, with free access, for the assessment of clinical reasoning in speech-language therapy, of students or young clinicians, with general practitioner training.

## METHOD

This study was approved by the Research Ethics Committee (COEP) of the Federal University of Minas Gerais (UFMG) under opinion number 5,824,852. All participants signed the Informed Consent Form – ICF. This is an instrument content validation study that follows the standards of the Joint Committee on Standards for Educational and Psychological Testing^(25)^ and the preparation of the SCT^(14-16)^.

### Stage I – construction of the instrument

The initial phase involved the development of *FonoTCS* cases and items. Six areas of speech-language pathology knowledge were selected in two domains (Chart 1).

**Chart 1:** Description of the areas of knowledge and domains covered by the *FonoTCS* questions

**Area**	**Assessment/diagnosis**	**Treatment/intervention**
**Audiology**	3	3
**Dysphagia**	1	1
**Language**	3	3
**Orofacial motricity**	3	1
**Public Health**	1	5
**Voice**	2	4
**Total**	13	17

**Figure 1:**
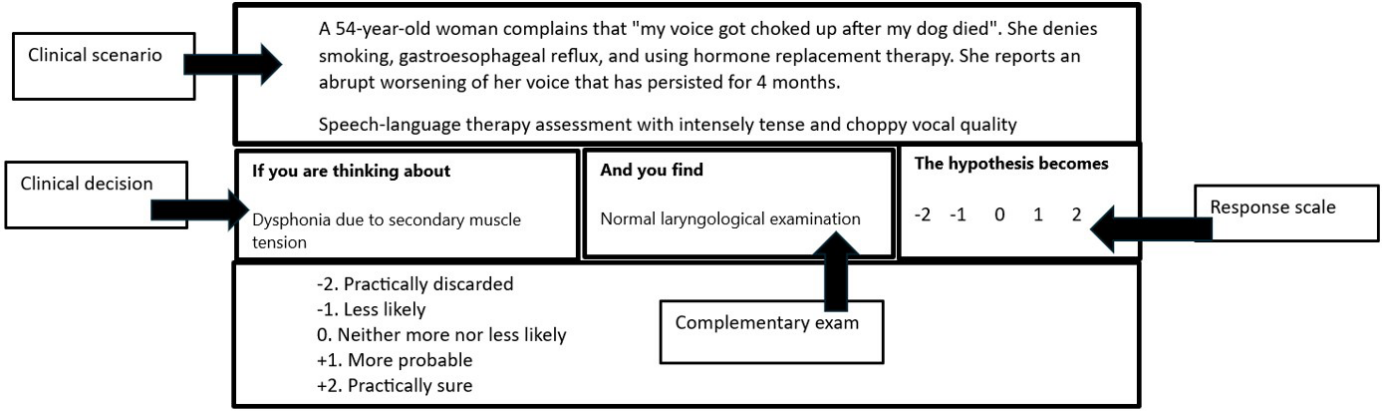
SCT question for speech-language therapy students in the area of voice

The *FonoTCS* was structured with 30 clinical cases in scenarios that incorporated uncertainty^(15)^. For each clinical case, four items were developed and presented in three parts. The first part (“if you are thinking about”) contains a relevant clinical decision. The second part (“and you find”) shows new information such as a sign or symptom, a physical or social condition that affects health, an imaging diagnosis, or the result of an exam/test. The third part (“the hypothesis becomes”) is a five-point Likert scale that captures examined users' decisions^(14)^ (Figure 1).

The examined users' task is to determine the impact of the discovery (second part) on the clinical decision (first part), in terms of direction (positive, negative, or neutral) and intensity (third part). The use of a Likert scale is based on script theory, which assumes that clinical reasoning is composed of a series of qualitative judgments^(26)^.

The *FonoTCS* presented, in its original version, 30 questions, that is, 30 clinical cases with four items each (120 items). Figure 1 shows an example of a question in the voice area with an item in the diagnosis domain.

A panel of experts developed the 30 questions, each accompanied by their clinical cases and items. The eligibility criteria for composing the panel were: being a speech-language therapist with clinical experience for more than 10 years, with a doctorate, and teaching at a Higher Education Institution (HEI). The experts were five speech-language therapists with more than 13 years of professional experience (mean=24.8, SD=7.5), and aged between 37 and 54 years (mean=48.2, SD=7.1), who had a consensus during the instrument construction process. Creating the questions involved holding three meetings, in which experts discussed challenging clinical cases and the items associated with them.

To prepare the questions (cases and items), the panel of experts followed these guidelines^(14,27)^: (i) describe everyday clinical scenarios that contain an element of uncertainty; (ii) specify each scenario: a) relevant hypotheses, investigation strategies or treatment options; b) the questions they ask when taking the patient's history, the signs they look for during different exams, and the tests they order to solve the problem; and c) clinical information, positive or negative, that they would look for in these investigations^(27)^; (iii) prepare the items of a clinical case within the same domain (diagnosis or treatment), to guarantee independence between the successive items of the clinical case, ensuring the principles of construction of the SCT^(14)^.

### Stage II - content validity

At this stage, the *FonoTCS* questions were distributed electronically to 15 evaluators, with a 30-day deadline for feedback. Speech-language therapists with a minimum master's degree and at least 10 years of generalist clinical experience were invited.

The assessments were carried out individually and independently. The judges used criteria of clarity, ethics, and relevance to evaluate the questions, using a Likert-type scale with five graduated points. On this scale, a value of 5 corresponded to “totally agree”, while a value of 1 corresponded to “totally disagree”.

Five different statements based on literature^(28)^ were presented to the judges. For the clarity of the questions, the judges evaluated two statements: 1) “This question is formulated precisely, without flaws or ambiguities”; and 2) “The question items present coherent and plausible clinical situations.” Regarding the relevance of the questions, the evaluators analyzed two statements: 1) “This question is relevant for the construction of the clinical reasoning of a speech-language therapist”; and 2) “This question shows a challenging clinical problem but appropriate for the level of knowledge of an inexperienced speech-language pathologist.” To evaluate the ethical issue, the following statement was used: “This question has adequate content in ethical, racial, and cultural terms.”

In addition to evaluations based on these statements, at the end of each question, evaluators could offer qualitative feedback, aiming to improve the quality of clinical scenarios and items.

To analyze the judges' responses, the Corrected Content Validity Coefficient (CVCc)^(29)^ of all five statements was calculated. Questions that presented a percentage of agreement equal to or lower than 80%^(29)^ for any of the statements evaluated by the judges were reviewed. In this case, the qualitative feedback from the judges was also analyzed to analyze the comments provided to understand the reasons behind the evaluations.

### Stage III - review of the question content

For the questions with the least agreement^(29)^ on any of the five statements, the group of five experts (Stage I) met to evaluate the clinical scenarios and items, and the judges' feedback. Based on the assessments and discussions, the cases and their items could be reformulated, restructured, or eliminated. All decisions were made by consensus, in a single meeting.

The criteria for reformulating the cases were those that needed to adapt the clarity of the clinical scenario and/or the items; to restructure the adjustment to the degree of clinical difficulty of the scenario and/or items; and eliminate cases considered to be of little relevance, redundant or with ethical problems.

## RESULTS

Thirteen (86.6%) of the speech-language therapists responsible for evaluating the content responded to the analysis. The judges were female, aged 34 to 46 years (mean=39.07, SD=4.11), eight (68%) with master´s degrees and five with a Ph.D. (32%), with generalist clinical practice that varied from 10 to 24 years (mean=15.38, SD=4.57).

Regarding the CVCc results, in the clarity criterion, question 1 presented agreement values that varied from 0.85 to 0.98 (mean=0.93, SD=0.035), and question 2 from 0.87 to 1.00 (mean=0.95, SD=0.031).

For the relevance criterion, question 1 had agreement values that varied from 0.89 to 1.00 (mean=0.98, SD=0.024), and question 2 from 0.78 to 0.98 (mean= 0.92, SD=0.05).

The agreement values on the ethics criterion question ranged from 0.97 to 1.00 (mean=0.99, SD=0.009).

In this version of the instrument, two (6.66%) *FonoTCS* cases presented scores (CVCc) of 0.78 and 0.80, both from the area of audiology in the evaluation/diagnosis domain, for question 2 related to the criterion of relevance.

Both were reviewed by experts (Stage 1) and restructured. The two clinical scenarios were more detailed, to make the case less uncertain and complex. One of the items was modified with the inclusion of more routine diagnostic information from the speech-language therapy clinic.

## DISCUSSION

Based on the theoretical model of clinical reasoning through script theory^(6-8)^, the SCT has three key characteristics for its elaboration: (1) respondents face uncertain clinical situations and must choose between several options found in their professional routine; (2) the response format reflects the way information is processed in challenging situations; and (3) the score takes into account the variability of experts' responses to different clinical situations^(30)^. The performance of the SCT as an assessment tool depends on the careful development of questions (cases and items) and the refined selection of experts in the construction and validation stages^(30)^.

According to the National Curricular Guidelines for the Undergraduate Course in Speech-Language Therapy (CNE/CES 5)^(9)^, the speech-language therapist needs to “have scientific, generalist clinical training, which allows them to master and integrate the knowledge, attitudes, and information necessary for the several types of speech-language therapy activities”^(9)^.

In this context, to construct the *FonoTCS* content, six areas of Speech-language Therapy knowledge were listed (audiology, language, orofacial motricity, dysphagia, voice, and collective health), in different life cycles and clinical environments (hospitals, clinics, offices, Basic Health Units), focusing on students and young speech-language therapists with generalist clinical training^(9)^. The objective of *FonoTCS* is to evaluate clinical reasoning in speech therapy at the end of graduation or the beginning of a professional career with the aim of, based on the results, promoting the improvement of clinical skills and decision-making based on speech therapy practice.

Therefore, the general principles for building the *FonoTCS* ^(30)^ were: **purpose** (formative assessment); **target group** (graduate students and young clinicians); and **knowledge domain** (speech-language therapy).

The guidelines for constructing the SCT^(14-16,30)^ were followed. Regarding the number of cases and items, 20 to 25 cases with three to four items per clinical case are recommended^(14,15,30)^. The *FonoTCS* was originally constructed with 30 cases with four items each (120 items), considering that in the analysis to evaluate the internal structure, around a quarter of the items will be removed^(15)^ after evaluating the psychometric properties of each of the items.

Five-point Likert scales are the most commonly used in the SCT^(17-20)^. The scale of responses generally ranges from -2 to +2 with a neutral point (0)^(14,15)^. The zero anchors on the scale, which relates to data that has no positive or negative impact on clinical decision-making, is not a refuge for candidates without an opinion, as it is not an easy task for a beginner to assert that a certain piece of clinical information does not have an impact on diagnostic or therapeutic decisions^(16)^. The three-point Likert scale (1, 0, +1) is recommended for developing SCT intended for learning tools^(15,30)^. The *FonoTCS* was developed with a five-point Likert scale, as it is an assessment instrument^(30)^.

At least four members are suggested to prepare the instrument^(14,15)^. Five members were invited to the *FonoTCS*, considering the training of these professionals within the areas of knowledge of speech-language therapy. All are doctors with teaching experience since both the degree of training^(30)^ and teaching practice^(15)^ are skills suggested by the SCT guidelines1^(4,15,30)^.

In the analysis of content validity, two questions (cases and items) were restructured. According to the judges' qualitative assessment (Stage 2), the cases presented complex clinical scenarios, which were not relevant for the assessment of graduating students and young clinicians. The group of experts (Stage 1) chose to add more information to the clinical scenarios and modify one of the items with more routine clinical diagnostic situations for the speech-language therapist, reducing the complexity of both questions.

The questions were maintained because the experts (Stage 1) understood that, in the process of validating the internal structure, cases and items can be removed^(15,16)^. From a psychometric point of view, the ideal SCT questions are those that generate a variability of responses grouped around a modal response^(15,16)^.

Content assessment was important to ensure that the *FonoTCS* accurately assesses clinical reasoning in speech-language pathology.

## CONCLUSION

The Speech-Language Therapy Script Agreement Test (*FonoTCS*), for evaluating the clinical reasoning of students and young clinicians with generalist practice, is a valid instrument from the point of view of content (clarity, relevance, and ethics).

## References

[1] Norman G (2005). Research in clinical reasoning: past history and current trends. Med Educ.

[2] Cerullo JASB, Cruz DALM (2010). Raciocínio clínico e pensamento crítico. Rev Lat Am Enfermagem.

[3] Custers EJ (2013). Medical education and cognitive continuum theory: an alternative perspective on medical problem solving and clinical reasoning. Acad Med.

[4] Beckie TM, Lowry LW, Barnett S (2001). Assessing critical thinking in baccalaureate nursing students: a longitudinal study. Holist Nurs Pract.

[5] Hoben K, Varley R, Cox R (2007). Clinical reasoning skills of speech and language therapy students. Int J Lang Commun Disord.

[6] Schmidt HG, Rikers RJ (2007). How Expertise develops in medicine: knowledge encapsulation and illness scripts formation. Med Educ.

[7] Peixoto JM, Santos SME, Faria RMD, Moura AS (2018). Clinical reasoning development in medical students. Rev Bras Educ Med.

[8] Schmidt HG, Mamede S (2015). How to improve the teaching of clinical reasoning: a narrative review and a proposal. Med Educ.

[9] Brasil (2002). Resolução CNE/CES nº 5, de 19 de fevereiro de 2002. Institui as Diretrizes Curriculares Nacionais do Curso de Graduação em Fonoaudiologia.

[10] Eva KW (2004). What every teacher needs to know about clinical reasoning. Med Educ.

[11] Lake S, Mclnnes RJ (2012). Exploring cognitive skill development in midwifery education. Nurse Educ Pract.

[12] Ginsberg SM, Friberg JC, Visconti CF (2016). Diagnostic reasoning by experienced speech-language pathologists and student clinicians. Commun Sci Disord.

[13] Deschênes MF, Charlin B, Gagnon R, Goudreau J (2011). Use of a script concordance test to assess development of clinical reasoning in nursing students. J Nurs Educ.

[14] Fournier JP, Demeester A, Charlin B (2008). Script concordance test: guidelines for construction. BMC Med Inform Decis Mak.

[15] Dory V, Gagnon R, Vanpee D, Charlin B (2012). How to construct and implement script concordance tests: insights from a systematic review. Med Educ.

[16] See KC, Tan KL, Lim TK (2014). The script concordance test for clinical reasoning: re-examining its utility and potential weakness. Med Educ.

[17] Kelkar AJ, Bhandary S, Chacko T (2022). Addressing the need to develop critical thinking skills in the new competency-based medical education post graduate curriculum in pathology: experience-sharing of the process of development and validation of script concordance test. AM&HS..

[18] Redmond C, Jayanth A, Beresford S, Carroll L, Johnston ANB (2022). Development and validation of a script concordance test to assess biosciences clinical easoning skills: A cross-sectional study of 1st year undergraduate nursing students. Nurse Educ Today.

[19] Vital S, Wulfman C, Girard F, Tamimi F, Charlin B, Ducret M (2020). Script concordance tests: a call for action in dental education. Eur J Dent Educ.

[20] Cobb KA, Brown G, Hammond R, Mossop LH (2015). Students’ perceptions of the script concordance test and its impact on their learning behavior: a mixed methods study. J Vet Med Educ.

[21] O’Brien SR, Dillon N, Linskey M, Lagueras K, Uhl J, Conroy S (2023). Initial validation of a script concordance test to measure the development of clinical reasoning among physical therapy residents. JCEPT.

[22] Piovezan RD, Custódio O, Cendoroglo MS, Batista NA (2010). Teste de Concordância de Scripts: uma proposta para a avaliação do raciocínio clínico em contextos de incerteza. Rev Bras Educ Med.

[23] Menezes SSC (2016). Avaliação do raciocínio clínico: adaptação e validação do Test de Concordance de Scripts Human Caring.

[24] Downing SM (2003). Validity: on meaningful interpretation of assessment data. Med Educ.

[25] American Educational Research Association (2014). The standards for educational and psychological testing.

[26] Gagnon R, Charlin B, Roy L, St-Martin M, Sauvé E, Boshuizen HPA (2006). The cognitive validity of the Script Concordance Test: a processing time study. Teach Learn Med.

[27] Charlin B, Roy L, Brailovsky C, Goulet F, van der Vleuten C (2000). The Script Concordance test: a tool to assess the reflective clinician. Teach Learn Med.

[28] Aldekhayel SA, Alselaim NA, Magzoub ME, Al-Qattan MM, Al-Namlah AM, Tamim H (2012). Constructing a question bank based on script concordance approach as a novel assessment methodology in surgical education. BMC Med Educ.

[29] Hernández-Nieto RA (2002). Contributions to statistical analysis.

[30] Lubarsky S, Dory VR, Duggan P, Gagnon R, Charlin B (2013). Script concordance testing: from theory to practice: AMEE Guide No. 75. Med Teach.

