# Supplementary material for: Test for clinical reasoning evaluation in Speech-Language Pathology: content validity
Source: Codas. 2024 May 31;36(4):e20230276. doi: 10.1590/2317-1782/20242023276en (PMC11189150; doi:10.1590/2317-1782/20242023276en)
Supplement: Translation

# Translation: Teste para avaliação do raciocínio clínico em Fonoaudiologia: validade de conteúdo

**DOI:** 10.1590/2317-1782/20242023276pt

**Authors:** Ana Cristina Côrtes Gama, Aline Mansueto Mourão, Adriane Mesquita Medeiros, Patrícia Cotta Mancini, Thais Helena Machado, Lara Gama Santos, Nayara Ribeiro Gomes

**Affiliations:** 1 Departamento de Fonoaudiologia, Faculdade de Medicina, Universidade Federal de Minas Gerais – UFMG - Belo Horizonte (MG), Brasil.; 2 Centro Federal de Educação Tecnológica de Minas Gerais – CEFET - Belo Horizonte (MG), Brasil.; 3 Programa de Pós-graduação (doutorado) em Ciências Fonoaudiológicas, Departamento de Fonoaudiologia, Faculdade de Medicina, Universidade Federal de Minas Gerais – UFMG - Belo Horizonte (MG), Brasil.

**Keywords:** Fonoaudiologia, Raciocínio Clínico, Diagnóstico Clínico, Tomada de Decisão Clínica, Estudantes, Aprendizagem

## Abstract

**Objetivo:**

validar o conteúdo do Teste de Concordância de Scripts em Fonoaudiologia, denominado FonoTCS.

**Método:**

Trata-se de estudo de validação de conteúdo de instrumento. Participaram da construção do FonoTCS cinco fonoaudiólogas, doutoras e docentes, com média de 24,8 anos de atuação profissional, que chegaram a um consenso durante o processo de construção do teste. Elaborou-se 30 questões e 120 itens contemplando as áreas de atuação fonoaudiológica. Em seguida, 15 fonoaudiólogas com titulação mínima de mestre, e com, no mínimo, 10 anos de atuação clínica generalista receberam eletronicamente o FonoTCS para validação de conteúdo por meio de questionário sobre critérios de clareza, ética e pertinência do conteúdo das questões. Para a análise das respostas calculou-se o Coeficiente de Validade de Conteúdo Corrigido de todas as afirmativas. Foram revisadas as questões com porcentagem de concordância igual ou inferior a 80%.

**Resultados:**

13 avaliadores responderam a análise, todos do sexo feminino, com média de idade de 39,07 anos, sendo oito mestres e cinco doutoras, com atuação clínica generalista média de 15,38 anos. Os valores médios do Coeficiente de Validade de Conteúdo Corrigido foram 0,93 e 0,95 para o critério de clareza; 0,98 e 0,92 para o critério de pertinência; e 0,99 para o critério de ética. Duas questões apresentaram notas de 0,78 e 0,80, sendo ambas da área de audiologia no domínio de avaliação/diagnóstico, para a questão relacionada ao critério de pertinência, sendo revisadas e reestruturadas pelos juízes.

**Conclusão:**

O FonoTCS é um instrumento válido do ponto de vista do conteúdo.

## INTRODUÇÃO

Conceitua-se raciocínio clínico como um processo cognitivo que permite ao profissional da saúde definir um correto diagnóstico e uma adequada conduta terapêutica diante de um determinado caso clínico^(1)^. O processo cognitivo envolvido compreende elaborações mentais ligadas ao atendimento dos usuários dos sistemas de saúde, e é um tema central nos campos da educação em saúde e no exercício da prática profissional^(2)^. Atualmente, uma das teorias aceitas para o raciocínio clínico, leva em consideração o desenvolvimento de *scripts* mentais^(3-6)^.

Nesta teoria, os profissionais da saúde, a partir da exposição repetitiva a casos clínicos, criam esquemas mentais de determinadas condições clínicas, chamados de “*script* de doença”, que ficariam armazenados na memória^(7)^. Um *script* representaria, portanto, uma rede de conhecimento específico, onde múltiplos elementos de informações estão organizados de acordo com suas relações^(6)^.

Profissionais experientes possuem redes elaboradas de conhecimento ligadas ao processo diagnóstico ou de decisão terapêutica, ou seja, *scripts* mentais refinados, que proporcionam um raciocínio clínico assertivo^(7)^. Os *scripts* são feitos correlacionando informações dos distúrbios ou doenças, suas características clínicas e possibilidades de tratamento^(6,8)^.

Segundo as Diretrizes Curriculares Nacionais do Curso de Graduação em Fonoaudiologia (CNE/CES 5)^(9)^, uma das competências necessárias para o exercício da profissão é a capacidade de tomada de decisões para as práticas fonoaudiológicas, que envolvem o correto diagnóstico e a definição do modelo de intervenção mais adequado a cada problema clínico^(9)^.

Estratégias de aprendizagem para o desenvolvimento do raciocínio clínico^(10)^ são descritas, além de formas para avaliar e acompanhar o progresso do desempenho de estudantes da área da saúde^(11)^.

Apontam-se dificuldades de raciocínio clínico de graduandos de Fonoaudiologia ao fazerem diagnósticos^(5)^ e diferenças importantes de raciocínio clínico entre estudantes e profissionais experientes^(12)^. O desafio de como avaliar o desempenho do raciocínio clínico dos estudantes durante a sua formação acadêmica é constante nos currículos da área da saúde, incluindo a fonoaudiologia^(13)^.

O teste de concordância de *script* (TCS) (*Script Concordance Test* - SCT) é baseado no princípio de que múltiplos julgamentos feitos no processo de raciocínio clínico podem ser investigados, e sua concordância com os de um painel de especialistas de referência pode ser medida^(14,15)^.

O TCS foi desenvolvido para avaliar o raciocínio em situações incertas^(16)^ que ocorrem frequentemente na prática diária, especialmente para os profissionais da saúde^(14)^. Diretrizes para a elaboração do TCS^(14-16)^ que explicita regras para a preparação e administração dos protocolos, foram propostas, para que estes instrumentos sejam confiáveis e válidos. O princípio do teste é ser baseado em casos clínicos que devem ser descritos em cenários curtos e sempre incorporar incerteza^(15)^.

Como diretrizes para a construção do TCS algumas recomendações são apresentadas^(14-16)^ como: número de casos; número de membros para a elaboração do instrumento; análise da validade de conteúdo; definição da pontuação do teste a partir do método de escore agregado; e apresentação no formato eletrônico para apresentar exames por imagem e devolutiva dos resultados aos examinandos.

Uma série de estudos sobre o TCS foram desenvolvidos nas áreas de medicina^(17)^, enfermagem^(18)^, odontologia^(19)^, veterinária^(20)^, e na fisioterapia^(21)^. Os resultados apontaram para a validade do TCS e sua capacidade em diferenciar o raciocínio em função do grau de experiência profissional^(13,17-19)^. No Brasil o TCS foi desenvolvido para a avaliação do raciocínio clínico de estudantes de medicina em situações clínicas de geriatria^(22)^, e na enfermagem^(23)^. Não encontramos na literatura estudos que propõe o TCS na fonoaudiologia.

Instrumentos de avaliação desempenham um papel crucial na obtenção de informações. Contudo, essas ferramentas devem exibir atributos psicométricos, como validade e confiabilidade, a fim de assegurar a confiança nos indicadores que estão sendo avaliados^(24)^. O *Joint Committee on Standards for Educational and Psychological Testing* ^(25)^ apresenta cinco fontes de evidência de validade: (1) conteúdo; (2) estrutura interna; (3) relação com medidas externas; (4) padrão de resposta aos itens; e (5) consequencial^(25)^.

Considerando que instrumentos sobre avaliação do raciocínio clínico são incipientes, e que o TCS foi validado para estudantes de diversas áreas^(17-21)^, e que para a fonoaudiologia ainda não existem pesquisas sobre o TCS, este projeto objetiva validar o conteúdo do Teste de Concordância de *Script* em Fonoaudiologia, denominado FonoTCS.

A partir dos resultados desta pesquisa, o FonoTCS passará para a validação da estrutura interna e o desenvolvimento de seu formato virtual, com acesso livre, para a avaliação do raciocínio clínico em fonoaudiologia, do estudante ou jovem clínico, com formação generalista.

## MÉTODO

O presente estudo foi aprovado pelo Comitê de Ética em Pesquisa (COEP) da Universidade Federal de Minas Gerais (UFMG) sob parecer número 5.824.852. Todos os participantes assinaram o Termo de Consentimento Livre e Esclarecido – TCLE. Trata-se de estudo de validação de conteúdo de instrumento que segue as normas do *Joint Committee on Standards for Educational and Psychological Testing* ^(25)^ e de elaboração do TCS^(14-16)^.

### Etapa I – construção do instrumento

A fase inicial envolveu a elaboração dos casos e itens do FonoTCS. Foram selecionadas seis áreas de conhecimento fonoaudiológico em dois domínios (Quadro 1).

**Quadro 1:** Descrição das áreas de conhecimento e dos domínios contemplados pelas questões do FonoTCS

**Área**	**Avaliação/diagnóstico**	**Tratamento/intervenção**
**Audiologia**	3	3
**Disfagia**	1	1
**Linguagem**	3	3
**Motricidade orofacial**	3	1
**Saúde Coletiva**	1	5
**Voz**	2	4
**Total**	13	17

O FonoTCS foi estruturado com 30 casos clínicos em cenários que incorporaram incerteza^(15)^. Para cada caso clínico foram desenvolvidos quatro itens, apresentados em três partes. A primeira parte ("se você está pensando em") contém uma decisão clínica relevante. A segunda parte ("e você encontra") apresenta uma nova informação, como um sinal ou sintoma, uma condição física ou social que afeta a saúde, um diagnóstico de imagem ou resultado de um exame/teste. A terceira parte ("a hipótese se torna") é uma escala Likert de cinco pontos que captura decisões dos examinandos^(14)^ (Figura 1).

**Figura 1:** Questão do TCS para estudantes de fonoaudiologia na área de voz

A tarefa dos examinandos é determinar o impacto da nova descoberta (segunda parte) na decisão clínica (primeira parte), em termos de direção (positiva, negativa ou neutra) e intensidade (terceira parte). A utilização de uma escala Likert se fundamenta na teoria de *script*, que pressupõe que o raciocínio clínico é composto por uma série de julgamentos qualitativos^(26)^.

O FonoTCS apresentou, em sua versão original, 30 questões, ou seja, 30 casos clínicos com quatro itens cada (120 itens). A Figura 1 apresenta um exemplo de questão na área de voz com um item no domínio de diagnóstico.

As 30 questões, cada uma acompanhada de seus casos clínicos e itens, foram desenvolvidas por um painel de experts. Foram critérios de elegibilidade para compor o painel, ser fonoaudiólogo com atuação clínica há mais de 10 anos, com título de doutor e docente em Instituição de Ensino Superior (IES). Os experts foram cinco fonoaudiólogas com mais de 13 anos de atuação profissional (média=24,8, DP=7,5), e idade de 37 a 54 anos (média=48,2, DP= 7,1), que chegaram a um consenso durante o processo de construção do instrumento. A criação das questões envolveu a realização de três reuniões, durante as quais os especialistas debateram casos clínicos desafiadores e os itens associados a eles.

Para a elaboração das questões (casos e itens), o painel de experts seguiu as seguintes orientações^(14,27)^: (i) descrever cenários clínicos cotidianos que contenham um elemento de incerteza; (ii) especificar para cada cenário: a) hipóteses relevantes, estratégias de investigação ou opções de tratamento; b) as perguntas que fazem ao coletar a história do paciente, os sinais que procuram durante diferentes exames, e os testes que solicitam para resolver o problema; e c) informações clínicas, positivas ou negativas, que procurariam nessas investigações^(27)^; (iii) elaborar os itens de um caso clínico dentro do mesmo domínio (diagnóstico ou tratamento), para garantir independência entre os sucessivos itens do caso clínico, assegurando os princípios da construção do TCS^(14)^.

### Etapa II - validade de conteúdo

Nesta etapa, as questões do FonoTCS foram distribuídas eletronicamente a 15 avaliadores, com um prazo de 30 dias estabelecido para o retorno. Foram convidados fonoaudiólogos com titulação mínima de mestre, e com, no mínimo, 10 anos de atuação clínica generalista.

As avaliações foram realizadas de forma individual e independente. Os juízes utilizaram critérios de clareza, ética e pertinência para avaliar as questões, empregando uma escala do tipo Likert com cinco pontos graduados. Nessa escala, o valor 5 indicava "concordo totalmente", enquanto o valor 1 correspondia a "discordo totalmente".

Foram apresentadas aos juízes cinco declarações distintas baseadas na literatura^(28)^. No contexto da clareza das questões os juízes avaliaram duas afirmações: 1) "Esta questão está formulada de maneira precisa, sem falhas ou ambiguidades"; e 2) "Os itens da questão apresentam situações clínicas coerentes e plausíveis". Em relação à pertinência das questões, os avaliadores analisaram duas afirmações: 1) "Esta questão é relevante para a construção do raciocínio clínico de um profissional fonoaudiólogo"; e 2) "Esta questão apresenta um problema clínico desafiador, mas apropriado para o nível de conhecimento de um fonoaudiólogo inexperiente". Para a avaliação da questão ética, foi utilizada a seguinte declaração: "Esta questão apresenta conteúdo adequado em termos éticos, raciais e culturais".

Além das avaliações baseadas nessas afirmações, ao final de cada questão, os avaliadores podiam oferecer uma devolutiva qualitativa, visando aprimorar a qualidade dos cenários clínicos e itens.

Para a análise das respostas dos juízes, foi calculado o Coeficiente de Validade de Conteúdo Corrigido (CVCc)^(29)^ de todas as cinco afirmativas. Foram revisadas as questões que apresentaram uma porcentagem de concordância igual ou inferior a 80%^(29)^ para qualquer uma das afirmativas avaliadas pelos juízes. Neste caso, foi também analisada a devolutiva qualitativa dos juízes para análise dos comentários fornecidos para entender os motivos por trás das avaliações.

### Etapa III - revisão de conteúdo das questões

Para as questões menos concordantes^(29)^ em qualquer uma das cinco afirmativas, o grupo de cinco experts (Etapa I) se reuniu para avaliar os cenários clínicos e itens, e as devolutivas dos juízes. Com base nas avaliações e discussões, os casos e seus itens poderiam ser reformulados, reestruturados ou eliminados. Todas as decisões foram realizadas por consenso, em uma única reunião.

Foram critérios para reformular os casos aqueles que necessitassem adequar a clareza do cenário clínico e/ou dos itens; para reestruturação a adequação no grau de dificuldade clínica do cenário e/ou dos itens; e a eliminação os casos considerados pouco relevantes, redundantes ou com problemas éticos.

## RESULTADOS

Dos fonoaudiólogos responsáveis pela avaliação do conteúdo, 13 (86,6%) responderam a análise. Os juízes eram do sexo feminino, com idade de 34 a 46 anos (média=39,07, DP=4,11), sendo oito (68%) mestres e cinco (32%) doutoras, com atuação clínica generalista que variou de 10 a 24 anos (média=15,38, DP=4,57).

Com relação aos resultados do CVCc, no critério de clareza, a questão 1 apresentou valores de concordância que variaram de 0,85 a 0,98 (média=0,93, DP=0,035), e a questão 2 de 0,87 a 1,00 (média=0,95, DP=0,031).

Para o critério de pertinência, a questão 1 apresentou valores de concordância que variaram de 0,89 a 1,00 (média=0,98, DP=0,024), e a questão 2 de 0,78 a 0,98 (média=0,92, DP=0,05).

Os valores de concordância na questão do critério de ética foram de 0,97 a 1,00 (média=0,99, DP=0,009).

Nesta versão do instrumento, dois (6,66%) casos do FonoTCS apresentaram notas (CVCc) de 0,78 e 0,80, sendo ambos da área de audiologia no domínio de avaliação/diagnóstico, para a questão 2 relacionada ao critério de pertinência.

Ambos foram revisados pelos especialistas (Etapa 1) e reestruturados. Os dois cenários clínicos foram mais detalhados, com o intuito de deixar o caso menos incerto e complexo, e um dos itens foi modificado com a inclusão de informações diagnósticas mais rotineiras da clínica fonoaudiológica.

## DISCUSSÃO

Baseado no modelo teórico de raciocínio clínico pela teoria do *script* ^(6-8)^, o TCS possui três características-chave para sua elaboração: (1) os respondentes enfrentam situações clínicas incertas e devem escolher entre várias opções encontradas na rotina profissional; (2) o formato de resposta reflete a maneira como as informações são processadas em situações desafiadoras; e (3) a pontuação leva em consideração a variabilidade das respostas de especialistas a situações clínicas diversas^(30)^. O desempenho do TCS como ferramenta de avaliação depende do desenvolvimento cuidadoso das questões (casos e itens) e da seleção refinada de especialistas nas etapas de construção e validação^(30)^.

Segundo as Diretrizes Curriculares Nacionais do Curso de Graduação em Fonoaudiologia (CNE/CES 5)^(9)^ o fonoaudiólogo necessita “possuir uma formação científica, generalista, que permita dominar e integrar os conhecimentos, atitudes e informações necessários aos vários tipos de atuação em Fonoaudiologia”^(9)^.

Nesse contexto, para a construção do conteúdo do FonoTCS, elencou-se seis áreas de conhecimento da Fonoaudiologia (audiologia, linguagem, motricidade orofacial, disfagia, voz e saúde coletiva), nos diferentes ciclos de vida e ambientes clínicos (hospitais, clínicas, consultórios, Unidades Básicas de Saúde), com foco em estudantes e em jovens fonoaudiólogos com formação generalista^(9)^. O objetivo do FonoTCS é avaliar o raciocínio clínico em fonoaudiologia ao final da graduação ou no início da carreira profissional com o intuito de, a partir dos resultados, promover o aprimoramento das competências clínicas e a tomada de decisões embasadas na prática fonoaudiológica.

Portanto, os princípios gerais para a construção do FonoTCS^(30)^ foram: **propósito** (avaliação formativa); **grupo-alvo** (estudantes concluintes do curso de graduação e jovens clínicos); e **domínio** **de conhecimento** (fonoaudiologia).

As diretrizes para a construção do TCS^(14-16,30)^ foram seguidas. Com relação ao número de casos e itens, recomenda-se 20 a 25 casos com três a quatro itens por caso clínico^(14,15,30)^. O FonoTCS foi originalmente construído com 30 casos com quatro itens cada (120 itens), considerando que na análise de avaliação da estrutura interna, cerca de um quarto dos itens serão retirados^(15)^ após a avaliação das propriedades psicométricas de cada um dos itens.

As escalas do tipo Likert de cinco pontos são as mais comumente usadas no TCS^(17-20)^. A graduação das respostas geralmente varia de -2 a +2 com um ponto neutro (0)^(14,15)^. O âncora zero na escala, que se relaciona a dados que não têm impacto positivo ou negativo na tomada de decisão clínica, não é um refúgio para candidatos sem opinião, já que não é uma tarefa fácil para um iniciante afirmar que uma determinada informação clínica não tem impacto na decisão diagnóstica ou terapêutica^(16)^. A escala Likert de três pontos (1, 0, +1) são indicadas para a elaboração de TCS destinados a ferramentas de aprendizagem^(15,30)^. O FonoTCS foi elaborado com uma escala Likert de cinco pontos, por se tratar de um instrumento de avaliação^(30)^.

Indica-se pelo menos quatro membros para a elaboração do instrumento^(14,15)^. Para o FonoTCS foram convocados cinco membros, considerando a formação destes profissionais dentro das áreas de conhecimento da fonoaudiologia. Todos são doutores com experiência docente, já que tanto o grau de formação^(30)^, quanto a prática no ensino^(15)^ são competências sugeridas pelas diretrizes do TCS^(14,15,30)^.

Na análise da validade de conteúdo duas questões (casos e itens) foram reestruturadas. Segundo a avaliação qualitativa dos juízes (Etapa 2), os casos apresentavam cenários clínicos complexos, o que não era pertinente para a avaliação de estudantes concluintes e jovens clínicos. O grupo de especialistas (Etapa 1) optou por acrescentar mais informações nos cenários clínicos, e modificar um dos itens com situações clínicas de diagnóstico mais rotineiras do profissional fonoaudiólogo, diminuindo a complexidade de ambas as questões.

As questões foram mantidas porque os especialistas (Etapa 1) entenderam que, no processo de validação da estrutura interna, casos e itens podem ser retirados^(15,16)^. Do ponto de vista psicométrico, as perguntas do TCS ideais são aquelas que geram uma variabilidade de respostas agrupadas em torno de uma resposta modal^(15,16)^.

A avaliação de conteúdo foi crucial para garantir que o FonoTCS avalie de maneira precisa o raciocínio clínico em fonoaudiologia.

## CONCLUSÃO

O teste de concordância de *script* em fonoaudiologia (FonoTCS), para avaliação do raciocínio clínico de estudantes e jovens clínicos com atuação generalista, é um instrumento válido do ponto de vista do conteúdo (clareza, pertinência e ética).
